# Assessing the Validity of Normalizing Aflatoxin B_1_-Lysine Albumin Adduct Biomarker Measurements to Total Serum Albumin Concentration across Multiple Human Population Studies

**DOI:** 10.3390/toxins14030162

**Published:** 2022-02-23

**Authors:** Joshua W. Smith, Derek K. Ng, Christian S. Alvarez, Patricia A. Egner, Sean M. Burke, Jian-Guo Chen, Thomas W. Kensler, Jill Koshiol, Alvaro Rivera-Andrade, María F. Kroker-Lobos, Manuel Ramírez-Zea, Katherine A. McGlynn, John D. Groopman

**Affiliations:** 1 Department of Environmental Health and Engineering, Johns Hopkins Bloomberg School of Public Health, Baltimore, MD 21205, USA; joshuasmith@jhu.edu (J.W.S.); pegner1@jhu.edu (P.A.E.); sburke24@jhu.edu (S.M.B.); tkensler@fredhutch.org (T.W.K.); 2 Department of Epidemiology, Johns Hopkins Bloomberg School of Public Health, Baltimore, MD 21205, USA; dng@jhu.edu; 3 Division of Cancer Epidemiology and Genetics, National Cancer Institute, Rockville, MD 20850, USA; csalvarezp@gmail.com (C.S.A.); koshiolj@mail.nih.gov (J.K.); mcglynnk@mail.nih.gov (K.A.M.); 4 Department of Epidemiology, Qidong Liver Cancer Institute, Qidong 226200, China; chenjg@ntu.edu.cn; 5 Public Health Sciences Division, Fred Hutchinson Cancer Research Center, Seattle, WA 98109, USA; 6 Research Center for the Prevention of Chronic Diseases, Institute of Nutrition of Central America and Panama, Guatemala City 1188, Guatemala; arivera@incap.int (A.R.-A.); fkroker@incap.int (M.F.K.-L.); mramirez@incap.int (M.R.-Z.)

**Keywords:** aflatoxin, biomarker, albumin, adduct, normalization, dosimetry, mass spectrometry

## Abstract

The assessment of aflatoxin B_1_ (AFB_1_) exposure using isotope-dilution liquid chromatography-mass spectrometry (LCMS) of AFB_1_-lysine adducts in human serum albumin (HSA) has proven to be a highly productive strategy for the biomonitoring of AFB_1_ exposure. To compare samples across different individuals and settings, the conventional practice has involved the normalization of raw AFB_1_-lysine adduct concentrations (e.g., pg/mL serum or plasma) to the total circulating HSA concentration (e.g., pg/mg HSA). It is hypothesized that this practice corrects for technical error, between-person variance in HSA synthesis or AFB_1_ metabolism, and other factors. However, the validity of this hypothesis has been largely unexamined by empirical analysis. The objective of this work was to test the concept that HSA normalization of AFB_1_-lysine adduct concentrations effectively adjusts for biological and technical variance and improves AFB_1_ internal dose estimates. Using data from AFB_1_-lysine and HSA measurements in 763 subjects, in combination with regression and Monte Carlo simulation techniques, we found that HSA accounts for essentially none of the between-person variance in HSA-normalized (R^2^ = 0.04) or raw AFB_1_-lysine measurements (R^2^ = 0.0001), and that HSA normalization of AFB_1_-lysine levels with empirical HSA values does not reduce measurement error any better than does the use of simulated data (*n* = 20,000). These findings were robust across diverse populations (Guatemala, China, Chile), AFB_1_ exposures (10^5^ range), HSA assays (dye-binding and immunoassay), and disease states (healthy, gallstones, and gallbladder cancer). HSA normalization results in arithmetic transformation with the addition of technical error from the measurement of HSA. Combined with the added analysis time, cost, and sample consumption, these results suggest that it may be prudent to abandon the practice of normalizing adducts to HSA concentration when measuring any HSA adducts—not only AFB_1_-lys adducts—when using LCMS in serum/plasma.

## 1. Introduction

Aflatoxin B_1_ (AFB_1_) is a potent carcinogen that contaminates staple grains worldwide [1] and contributes to the global hepatocellular carcinoma burden [2]. Hepatic activation of AFB_1_ to its carcinogenic epoxide metabolite [3] results in the formation of mutagenic DNA adducts and adducts to lysine in albumin (AFB_1_-lys), both of which can be used as biomarkers of AFB_1_ exposure [4]. Quantitation of the AFB_1_-lys adduct biomarker using the current state-of-the-art isotope-dilution liquid chromatography-mass spectrometry (LCMS) analytical method [5] results in data expressed in units of volumetric concentration (e.g., pg AFB_1_-lys adduct/mL of serum or plasma). Conventionally, these values are transformed by dividing the measured AFB_1_-lys concentration by the concentration of total circulating human serum albumin (HSA), resulting in the quantitation of the AFB_1_-lys adduct relative to the total HSA concentration (e.g., pg AFB_1_-lys adduct/mg total HSA). This practice is grounded in prior analytical approaches for AFB_1_-lys adduct quantitation, such as immunoassays [6] or immunoaffinity purification of AFB_1_-lys from serum/plasma digests prior to quantification by liquid chromatography and fluorescence detection [7]. In both traditional methods, HSA precipitation or immunoaffinity purification of digested AFB_1_-lys increases assay sensitivity. However, since quantitated AFB_1_-lys values are no longer directly representative of the original volume of the sample from which they were derived (due to the precipitation or immunoaffinity purification), the values had to be normalized for assay input with a denominator other than the input volume. Thus, after the measurement of the total HSA concentration using one of the several available analytical approaches, AFB_1_-lys adduct values were normalized for assay input through their expression relative to the total HSA in the sample. These analytical methods are no longer the state-of-the-art approach for the measurement of AFB_1_-lys adducts, but the practice of normalization to HSA measured in a separate aliquot of serum (or plasma) has remained as a convention, including in previous reports from our group [8,9,10,11,12,13,14].

The purpose of this study was to assess whether the conventional practice of normalizing raw AFB_1_-lys adduct levels (expressed as a volumetric concentration, such as pg adduct/mL) to total HSA concentration (e.g., pg adduct/mg HSA) is warranted, given the added financial, labor, and sample consumption costs. Two approaches to assess this question could be: (1) to compare the internal dose values yielded by normalized vs. raw AFB_1_-lys adduct concentrations to measured levels of aflatoxin intake, or (2) to compare the predictive or prognostic value of normalized vs. raw AFB_1_-lys adduct values with disease incidence or outcomes in which aflatoxin plays an accepted etiological role. In regard to option 1, while our group has reported AFB_1_-lys adduct concentrations in humans with known levels of AFB_1_ intake [7,15], these analyses were performed using older AFB_1_-lys analytical methods rather than the current LCMS approach [5] and the samples are not available for re-analysis. Other groups have used the second approach, examining differences between HSA-normalized vs. raw AFB_1_-lys values within associations of AFB_1_ and hepatocellular carcinoma incidence [16]; however, the investigators utilized HSA precipitation prior to ELISA determination of AFB_1_-lys adduct levels, such that the total HSA measurement was used for normalization of the assay input. Additionally, while AFB_1_-lys measurement was conducted in samples from individuals, the association with hepatocellular carcinoma incidence was limited to ecological analysis, limiting the precision of the results.

In this report, we used a simulation approach to determine the efficacy of HSA normalization of AFB_1_-lys values determined by LCMS. To do so, we have paired a dataset of AFB_1_-lys adduct and HSA concentrations from nearly 800 persons with analysis by Monte Carlo simulation and regression, to interrogate whether normalization of AFB_1_-lys adduct levels to total HSA performs better than chance—independent of HSA assay format, study population, longitudinal changes in aflatoxin exposure, or the presence of hepatobiliary disease. Consistency in inferences between simulated and empirical data would suggest that the standardization of AFB_1_-lys values to HSA is not necessary and that it only reflects random error that is not associated with biological processes or technical variance.

## 2. Results

### 2.1. Normalization of AFB_1_-Lys Adduct Concentrations to Total HSA Corrects Estimates of AFB_1_ Internal Dose No Better than Chance in Guatemalan and Chinese Adults

**Figure 1:**
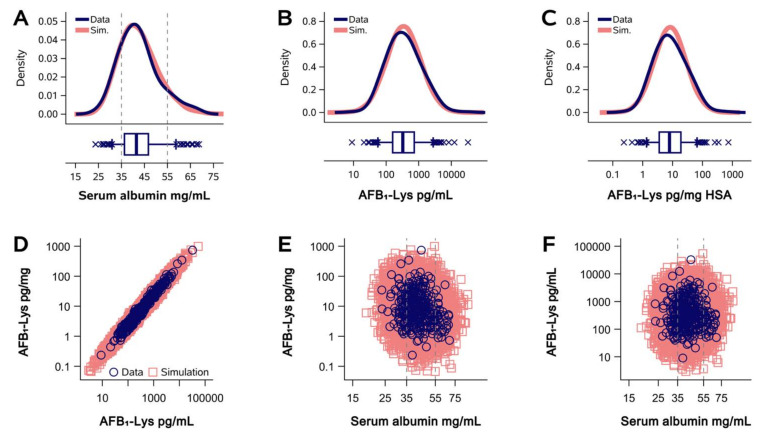

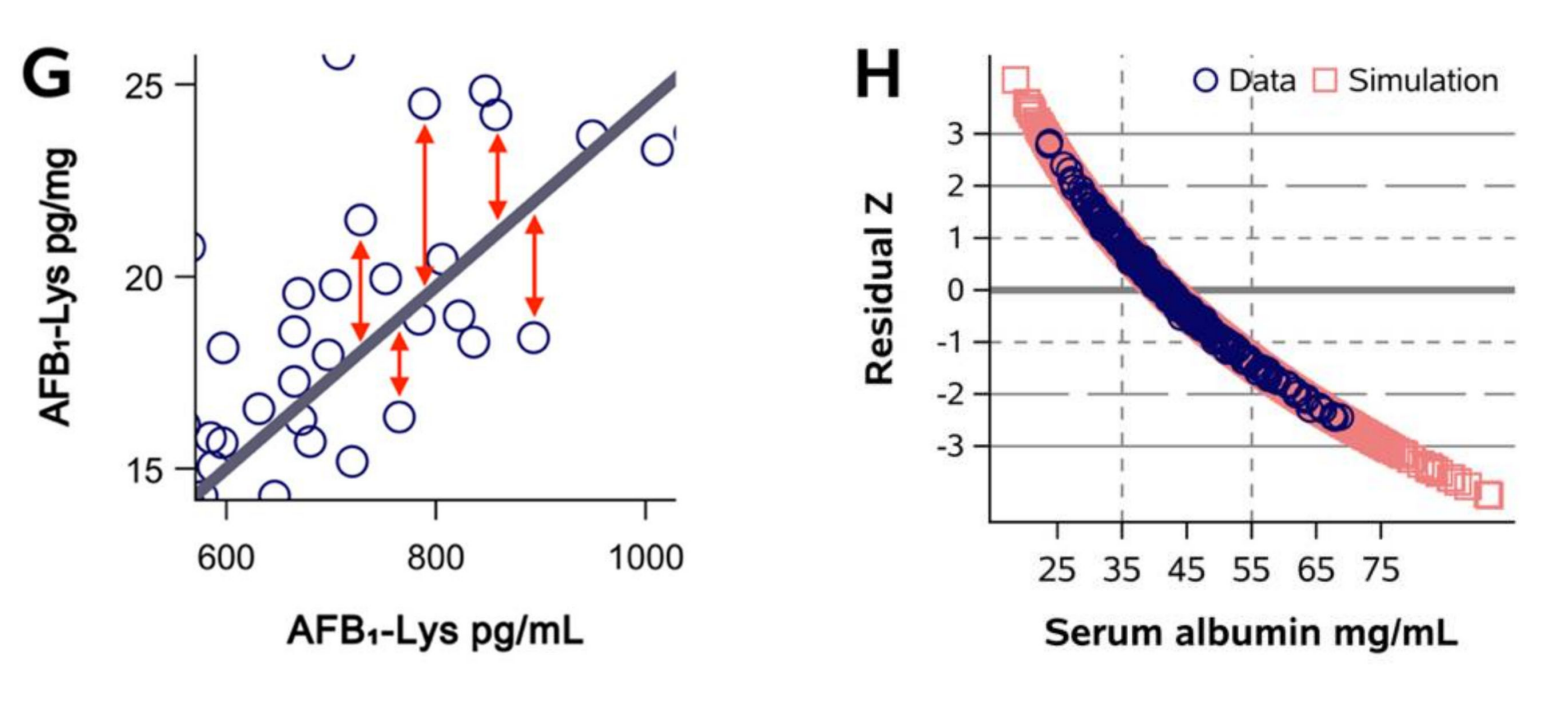
Guatemalan study (**A**–**C**), Density plots of HSA (**A**), AFB_1_-lys (**B**), and albumin-normalized AFB_1_-lys (**C**) concentrations in the empirically observed and simulated data. The dark blue tracing represents the empirically observed Guatemalan sample (*n* = 327); the coral tracing represents the simulated sample (*n* = 20,000). (**D**) Regression of AFB_1_-lys adducts as raw (pg/mL serum) vs. albumin-normalized values (pg/mg HSA) in empirically observed and simulated datasets. (**E**) Regression of HSA levels (mg/mL) vs. albumin-normalized values. (**F**) Regression of HSA levels vs. raw AFB_1_-lys concentrations. (**G**) Subset of observations from empirical data regression analysis in (**D**), red arrows denote regression residuals and illustrate the impact of albumin normalization on units of the AFB_1_ internal dose. (**H**) Standardized regression residuals (Z-scored Studentized residuals) vs. serum total HSA concentration. Vertical dashed lines in (**A**,**E**,**F**,**H**) designate the reference range of HSA.

Initial studies compared the relative contribution of variance components derived from AFB_1_-lys (pg/mL) and total HSA (mg/mL) measurements, within normalized AFB_1_-lys (pg/mg HSA) values. To do so, using data from our previously published cross-sectional study of AFB_1_ exposure in Guatemalan adults [8], we constructed bivariate and multiple linear regression models in which the dependent variable, AFB_1_-lys normalized to HSA (pg AFB_1_-lys/mg HSA), was regressed against the components of that calculated value, i.e., raw AFB_1_-lys (pg/mL) or HSA (mg/mL). In addition to AFB_1_-lys and total HSA measurements (Figure 1A–C), this dataset also contains a variety of demographic, behavioral, and clinical assessments; those previously found to be significantly predictive of AFB_1_ exposure in this population [8] were included in the multiple regression models. In a bivariate model, as expected, raw AFB_1_-lys concentrations were significantly, positively, and strongly predictive of HSA-normalized AFB_1_-lys concentrations (log[pg/mg] = 1.009 × log[pg/mL] −1.643, *p* < 0.0001, R^2^ = 0.97, Figure 1D). Given the use of HSA as the denominator in the normalization of raw AFB_1_-lys levels, total HSA concentrations were, as expected, inversely and significantly associated with HSA-normalized AFB_1_-lys concentrations (log[pg/mg] = −1.343 × log[mg/mL HSA] + 3.103, *p* < 0.0001). However, despite the significant linear relationship and in contrast with raw AFB_1_-lys, total HSA was a very poor predictor of normalized AFB_1_-lys concentrations (R^2^ = 0.04, Figure 1E). HSA levels were not significantly associated with concentrations of the raw AFB_1_-lys biomarker (*p* = 0.31, R^2^ = 0.0001, Figure 1F). These relationships were essentially unchanged in multiple regression models after adding demographic predictors of aflatoxin exposure [8] (Table 1). Notably, male sex, residence location (urban or rural), and income quintile were all stronger predictors of raw AFB_1_-lys levels (pg/mL) than was total HSA. These results, while expected, demonstrate that HSA levels account for very little of the variance in AFB_1_ internal dose (whether in raw or HSA-normalized units), bringing into question whether the HSA normalization practice removes biological or technical variance, as proposed.

Next, we sought to assess whether the variance attributed to HSA in the above analysis was biologically informative (e.g., normalization to HSA accounts for between-subject differences in liver function), or whether it was non-informative (e.g., HSA levels do not accurately reflect between-subject differences in liver function, or normalization to HSA adds variance due to technical errors intrinsic to the HSA assay). To do so, we compared empirical data from the Guatemala cross-sectional study to a simulated data set with the same distribution parameters as the empirical data, but populated with “participants” assigned randomly generated AFB_1_-lys and HSA values (*n* = 20,000 simulated subjects). As seen in Figure 1A–C, the distributions of the empirical data and the simulated dataset were identical for measurements of HSA (Figure 1A), raw AFB_1_-lys concentrations (Figure 1B), and normalized AFB_1_-lys (Figure 1C). To isolate the contribution of HSA measurement within the normalized AFB_1_-lys values, we revisited the bivariate regression model in which raw AFB_1_-lys concentrations were used to predict normalized AFB_1_-lys values (Figure 1D). Thus, any deviation from a perfect regression fit (i.e., the regression residual) is due to the contribution of the HSA denominator within normalized AFB_1_-lys values (Figure 1G). The regression residuals (standardized as Z-scores) were then plotted against the HSA concentration recorded for that participant (Figure 1H), reflecting the relationship between HSA concentration and the “correction” to raw AFB_1_-lys values achieved by normalization to HSA. As seen in Figure 1H, the empirical dataset collected from Guatemala is indistinguishable from randomly simulated data, suggesting that the “correction” applied to raw AFB_1_-lys values through HSA normalization is no different than that which would arise by chance alone. 

**Figure 2:**
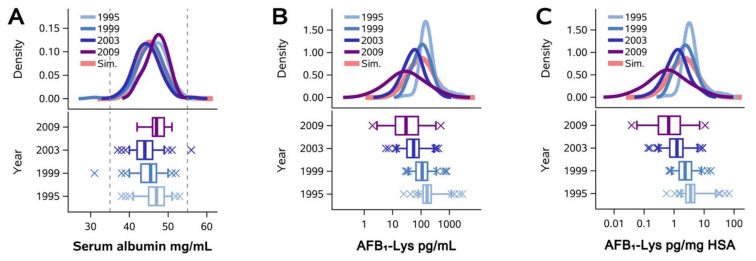

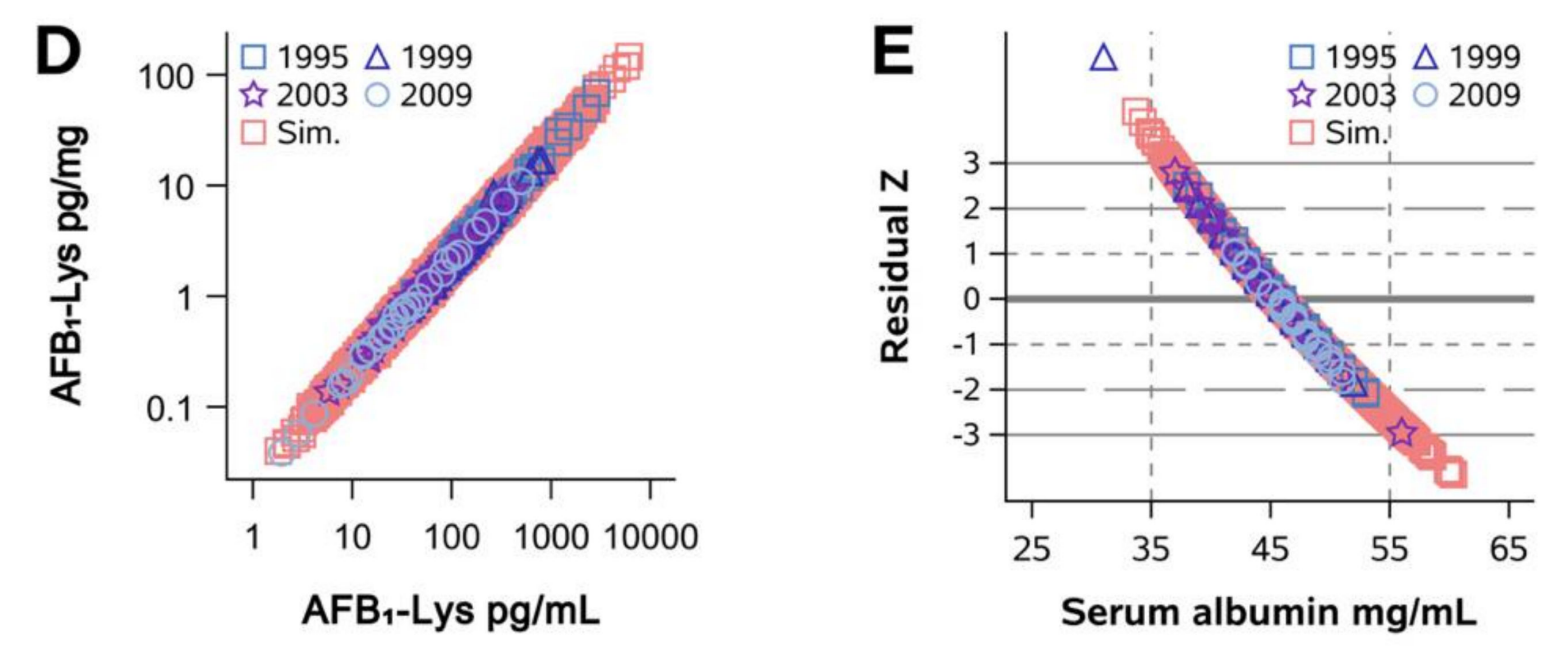
Qidong longitudinal study (**A**–**C**), Density plots of HSA (**A**), AFB_1_-lys (**B**), and albumin-normalized AFB_1_-lys (**C**) concentrations in the empirically observed and simulated data. Blue and purple tracings represent each of the four years in the empirically observed Chinese cohort (1995, *n* = 97; 1999, *n* = 92; 2003, *n* = 87; 2009, *n* = 30); coral tracings represent the simulated sample (*n* = 20,000). (**D**) Regression of AFB_1_-lys adducts as raw (pg/mL serum) vs. albumin-normalized values (pg/mg HSA) in empirically observed and simulated datasets. (**E**) Plot of standardized regression residuals from (**D**) vs. serum total HSA concentration. Vertical dashed lines in (**A**,**E**) designate the reference range of HSA.

We next tested these findings in a second population of adults with AFB_1_ exposure—residents of Qidong, Jiangsu Province, China. Previously [14], we have shown that AFB_1_-lys levels in residents of this community steadily decreased over a period of ~20 years, reflecting a dramatic shift from essentially universal AFB_1_ exposure in 1995 (98% detection prevalence) to the elimination of nearly all exposure by 2012 (7%). Thus, using these data (except for 2012, which did not have enough detectable samples), we attempted to replicate our findings from the Guatemalan study. As seen in Figure 2, as expected, we observed no trends in HSA levels from 1995–2009 (Figure 2A), but did observe temporally changing distributions of raw AFB_1_-lys (Figure 2B) and normalized AFB_1_-lys concentrations (Figure 2C). Simulated datasets reflected the distribution parameters for all years combined (coral tracings). As in Guatemala, measurements made in Chinese adults over a 14-year period of declining AFB_1_ exposure produced regression residuals (derived from the model in Figure 2D) that were indistinguishable from residuals generated by chance using the simulated dataset (Figure 2E).

### 2.2. Normalization of AFB_1_-Lys Adduct Concentrations to Total HSA Corrects Estimates of AFB_1_ Internal Dose No Better than Chance in Adults with Gallbladder Cancer

**Figure 3:**
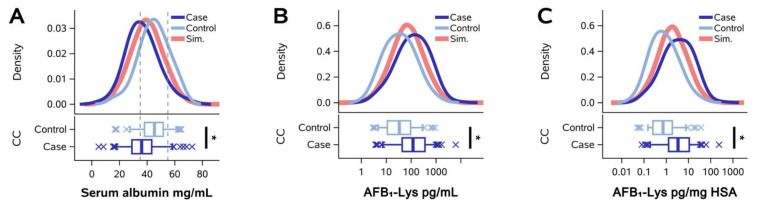

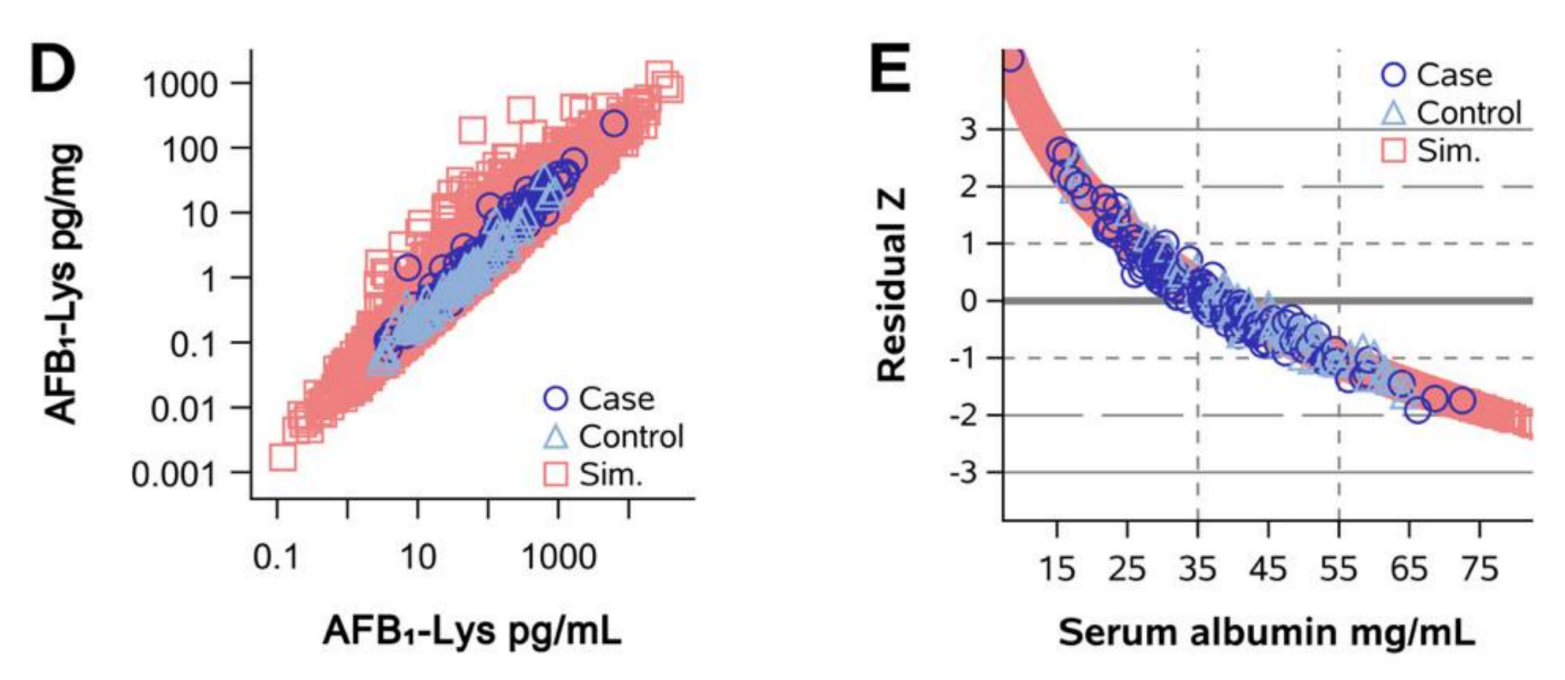
Gallbladder cancer case-control study (**A**–**C**), Density and boxplots of HSA (**A**), AFB_1_-lys (**B**), and albumin-normalized AFB_1_-lys (**C**) concentrations in the empirically observed and simulated data. Dark blue and light blue tracings represent persons with gallbladder cancer (Case; Chile *n* = 23, Shanghai *n* = 107) and controls, or persons with gallstones (Control; Chile *n* = 18, Shanghai *n* = 67), respectively. Coral tracings represent the simulated sample (*n* = 20,000). (**D**) Regression of AFB_1_-lys adducts as raw (pg/mL serum) vs. albumin-normalized values (pg/mg HSA) in empirically observed and simulated datasets. (**E**) Plot of standardized regression residuals from (**D**) vs. serum total HSA concentration. Vertical dashed lines in (**A**,**E**) designate the reference range of HSA.* *p* < 0.0001.

To assess whether HSA normalization of AFB_1_-lys adduct levels corrects for diminished protein synthesis or metabolic function in individuals with hepatobiliary disease, as has been proposed, we replicated our analysis using data from two published case-control studies, both of which reported a significantly increased risk of gallbladder cancer with elevated AFB_1_ exposure [11,12]. In line with these prior reports, the pooled analysis in the present study found that AFB_1_-lys adduct concentrations were significantly higher in gallbladder cancer cases than in controls or persons with gallstones, regardless of whether the values were untransformed (Figure 3B) or HSA-normalized (Figure 3C). However, circulating HSA exhibited an inverse relationship, such that HSA concentrations were significantly lower in gallbladder cancer cases than in controls (Figure 3A). Data for each of the two study sites (Chile and China) are shown separately in Supplemental Figure S1. As observed in Chinese and Guatemalan participants without hepatobiliary disease (Figure 1 and Figure 2), HSA normalization produced a distribution of regression residuals that were no different than those observed with simulated data (Figure 3D,E), again suggesting that normalization to empirically observed HSA concentrations reduces model variance no better than that achieved through normalization to randomly simulated values.

## 3. Discussion

In this study, we sought to test the hypothesis that the normalization of AFB_1_-lys adduct levels to total HSA concentrations does not improve the accuracy of internal dose estimates determined using the current state-of-the-art LCMS AFB_1_-lys assay. Our analysis attempted to quantify the effect of HSA normalization on the residual error present in AFB_1_-lys internal dose estimates. While the raw AFB_1_-lys adduct concentration is highly predictive of the HSA-normalized AFB_1_-lys value, there is error in the linear relationship between the two, such that the exact HSA-normalized AFB_1_-lys values cannot be predicted as a function of the raw AFB_1_-lys concentration alone (i.e., the R^2^ of the regression between raw and HSA-normalized AFB_1_-lys is not equal to 1). Since the HSA-normalized AFB_1_-lys value is, by definition, the quotient of error-prone estimates of raw AFB_1_-lys and HSA concentrations, this is to be expected. However, while the presence of error is to be expected, the sources and structures of the error can be informative and are worth further investigation. 

Although the true error is unobservable, it is approximated by the residuals of linear regression analysis: at a given level of the independent variable, the residual is the difference between an empirical observation of the dependent variable and the best-fit regression prediction of the dependent variable. Furthermore, although the residuals shown in Figure 1D are represented as a single error term in the linear regression equation ($y=\beta_{1}x+\beta_{0}+\varepsilon$), they receive contributions from multiple sources: technical (e.g., assay imprecision, operational mistakes, protease digestion efficiency, numerical rounding), biological (e.g., relationships between aflatoxin metabolism and HSA synthesis, between-person differences in the sample matrix composition, unaccounted confounding variables), and random error. Of course, these sources of variation within HSA-normalized AFB_1_-lys values are derived from the errors accompanying analytical estimates of both HSA and raw AFB_1_-lys adduct levels. However, in the case of the regression analysis in Figure 1D, the observed residuals can be attributed entirely to the HSA component of the HSA-normalized AFB_1_-lys value—if the dependent variable were also the raw AFB_1_-lys concentration instead of being the HSA-normalized AFB_1_-lys value, the mean squared error would be 0 and the R^2^ would equal 1. Thus, the regression analysis presented in Figure 1 (and subsequently, in other figures) isolates and reveals the contribution of HSA normalization to the error present in HSA-normalized AFB_1_-lys adduct estimates. 

The regression residuals are either informative (the magnitude, direction, and distributions collectively explain a portion of the technical and biological error in the measurements), are a result of statistical noise, or are a combination of the two. Under an assumption that HSA normalization adds information by systematically accounting for variation in hepatic function or other confounding variables, the regression residuals would reflect the gains in precision from the normalization operation. In other words, HSA normalization adds value if it is true that the raw AFB_1_-lys adduct concentration imprecisely reflects the AFB_1_ internal dose or AFB_1_-related disease risk, and that the adjustment made by HSA normalization minimizes error and moves the estimate closer to the unknown true value. Conversely, if the HSA concentration does not adequately account for or is entirely unassociated with error contributed by the confounding variable(s), then the regression residuals document a deleterious addition of error. This would result in a diminution in statistical power, a bias towards or away from the null hypothesis, or both. Clearly, distinguishing which case is more reflective of the truth is critical to the use of the AFB_1_-lys adduct biomarker going forward. 

One way to test this question is to compare relationships observed empirically in data from human samples with those that arise from randomly simulated values obtained from distributions with parameters equivalent to those in the empirical data. If the observed data are largely identical to those derived from simulated data, it can be deduced that the normalization of AFB_1_-lys to HSA is uninformative and adds error to estimates of exposure or disease risk. On the other hand, if the observed data deviate significantly from simulated distributions, then it can be assumed that biological mechanisms, or some other systematic source of error, account for this deviation and that HSA normalization is—to some degree—informative. 

Specifically, we focused on the relationship between HSA concentrations and the residuals from the linear regression of raw vs. HSA-normalized AFB_1_-lys adduct levels, since the residuals of this regression analysis isolate the variance derived from the HSA component of the HSA-normalized AFB_1_-lys value. However, we must first define the null hypothesis, where there exists no biological relationship between HSA concentrations and HSA-normalization regression residuals. In fact, this null hypothesis is presented by the simulated data sets, since simulated HSA and AFB_1_-lys values within each of the *n* = 20,000 “participants” per data set were randomly generated and paired to produce simulated HSA-normalized AFB_1_-lys values. As seen in Figure 1H, Figure 2E and Figure 3E, as the simulated HSA concentration decreases, the Z-scored regression residuals increase; the opposite occurs with increasing HSA levels. These patterns are to be expected in the randomly simulated data, given the internal arithmetic of HSA normalization. 

We now compare the null hypothesis above with the alternative hypothesis, formed on the assumption that the normalization of raw AFB_1_-lys to HSA is biologically valid and effective in reducing measurement error. If low HSA concentration reflects reduced hepatic metabolic capacity or a limited availability of protein substrate for the adduction of AFB_1_ to HSA, one could hypothesize that in the presence of low circulating HSA levels, raw AFB_1_-lys adduct concentration would underestimate the AFB_1_ internal dose. Thus, at constant levels of AFB_1_ exposure, AFB_1_-lys adduct concentrations would decrease in some proportion with HSA and normalization could be useful to correct for the underestimation. To support this hypothesis, it would be expected that, in contrast to simulated data, the empirically observed HSA-normalization regression residuals do not deviate greatly from zero at low concentrations of HSA, as this is the range where normalization is proposed to be most effective. Instead, we consistently observed the opposite—empirical residuals at HSA concentrations lower than the normal reference range (35–55 mg/mL) exhibit a monotonic increasing trend with decreasing HSA and demonstrate no inflection point where the hypothesized normalizing effect may occur. Additionally, aside from sample size, the residuals in the empirical data and the simulated data appear nearly identical—indicating that empirically determined HSA is no better than randomly simulated values for the purposes of reducing error in AFB_1_-lys adduct internal dose estimates. 

To assure ourselves that our initial results in the Guatemalan study were not idiosyncratic to a specific population, a particular method of HSA measurement, or a particular range of AFB_1_ exposures, we repeated our analyses with raw data from a previously published report in Qidong, People‘s Republic of China [14]. As shown in Figure 2A–C, between 1995 and 2009, while HSA concentrations remained within normal reference ranges, AFB_1_-lys adduct levels progressively decreased by approximately 80%; our original publication found that this was likely due to shifts in Chinese agro-economic policy [14] and that this diminished AFB_1_ exposure contributed significantly to a now 30-year trend of decreasing liver cancer mortality in Qidong [17]. While AFB_1_-lys adducts in this study were measured using the same method as that used in the Guatemalan study (using the same mass spectrometer and calibration standards), this Qidong ecological study utilized a different HSA assay (bromocresol purple vs. sandwich ELISA in the Guatemalan study), featured a different participant population, and exhibited a lower range of aflatoxin internal dose (52.4–165.4 pg/mL inter-quartile range in Qidong vs. 156.7–702.7 pg/mL in the Guatemala study). Despite the differences with the Guatemalan study, we replicated our earlier findings—across four screenings spanning 14 years, HSA was consistently no better of a normalizer for raw AFB_1_-lys than randomly simulated values. 

Finally, we wanted to test our approach in a context where AFB_1_ exposure may be concurrent with hepatobiliary disease, where decreases in hepatic metabolic capacity and HSA concentrations are etiologically linked. Specifically, we explored the use of HSA for normalization of raw AFB_1_-lys adduct levels in two case-control studies of gallbladder cancer. In contrast to the temporal stability of HSA concentrations in the longitudinal Qidong study, HSA concentrations were not equivalent between gallbladder cancer cases and controls—HSA levels in the cancer cases were significantly lower than in the controls (*p* < 0.0001), likely a result of hepatic injury secondary to bile duct obstruction or damage [18,19,20]. In the Guatemala and Qidong study participants, where hepatic function was likely within normal ranges, our data suggest that HSA normalization does not reduce error any better than chance. However, in the setting of gallbladder cancer, where the groups to be compared on the basis of AFB_1_-lys internal dose likely also differ in hepatic function, normalization with HSA as a surrogate for liver injury may be warranted. Nonetheless, we found that regression residuals from gallbladder cancer cases and controls were indistinguishable from one another and were no different than residuals from simulated data. Thus, these results indicate that HSA normalization does not reduce the error in AFB_1_-lys internal dose estimates in the setting of hepatic disease secondary to gallbladder cancer, but likely adds technical variance, reducing estimate precision and potentially increasing the risk of a type 2 error in statistical inference. 

While the HSA normalization approach has been considered conventional practice for reporting AFB_1_-lys adduct levels, the various justifications for its continued use do not withstand scrutiny. Fundamentally, all rationales cited in support of HSA normalization argue that the practice corrects for between-sample variation in HSA concentration and thus the pool of HSA from which the stoichiometrically rare HSA molecules carrying an AFB_1_-lys adduct are sampled. This variation in true HSA concentration within a given sample originates from either random chance during sampling of a volume of serum or plasma or from biological factors (between-person differences in hepatic metabolism of AFB_1_, HSA synthesis, etc.), and is added to the technical variation inherent in the assay used to produce estimates of HSA concentrations (assay precision). Utility of the HSA normalization approach thus depends on both effective correction for random and/or biological variance sources and low technical variance within the assay. 

With the current LCMS approach for AFB_1_-lys measurement, analysis is performed directly using serum/plasma samples rather than HSA precipitates or immunoaffinity purified fractions, as in prior methods [6,7]. Thus, the volume of the sample is precisely known and can serve as a valid denominator for quantitative estimates of internal dose, as with many other circulating biomarkers. Since calibrated pipettors dispense the 200 μL typically used in the LCMS assay at an accuracy within ±1% (ISO specification 8655-2:2002), random variance during sampling is not a substantial contributor to overall AFB_1_-lys measurement error and does not necessitate correction with HSA normalization. 

Additionally, the LCMS assay utilizes an isotopically labeled AFB_1_-lys internal standard, which corrects for analyte recovery by solid-phase extraction (SPE) during processing [5]. HSA assays used for normalization cannot address this source of technical variation within the LCMS assay, not only because SPE recovery variance is introduced after proteolytic digestion of HSA to single amino acid residues (which eliminates the capacity for HSA determination in the digested, extracted sample), but also because determinations of total HSA and AFB_1_-lys levels are not performed in the same aliquot of serum/plasma. Although measurement of HSA in a second aliquot clearly should produce values that are correlated with the concentration of HSA present in the aliquot used for LCMS analysis, this correlation between repeated measurements is subject to technical error within the HSA assay—error which can be substantial, depending on the approach used for HSA determination [21]. As a result, if the measurement of HSA in a second aliquot is to be used as an estimate of HSA concentrations in the aliquot used for LCMS AFB_1_-lys analysis, the utility of such an approach is dependent upon the technical variance within the HSA assay being substantially smaller than the true between-person variance in HSA concentration. Furthermore, samples exhibiting significant hemolysis can lead to aberrant HSA determinations, adding additional technical error [22,23]. In a recent publication in which we used a validated ELISA-based HSA assay [8], the percent coefficient of variation (%CV) for HSA concentrations across 400 persons (measured in technical duplicates) was 20.9%, while the inter-assay %CV for a pooled serum quality control sample was 17.2% across 15 assays (measured in technical duplicates in each assay). Thus, in the case of this validated HSA assay, technical variance from the HSA measurement itself appears to account for most of the between-person variance observed in humans. In comparison, in a recent study from our group, the average %CV for raw AFB_1_-lys adduct concentrations determined by LCMS in pooled serum quality control samples—measured at 3 separate levels in each of 35 separate analytical batches—was 10.8% [24]. Thus, HSA normalization does not appear to be an effective strategy to account for technical variance in aflatoxin internal dose estimates, as the HSA denominator likely contributes substantially more technical variance to the final estimate than does the AFB_1_-lys numerator.

Several rationales based on the following established physiological facts have been presented in support of HSA normalization of AFB_1_-lys adduct values: (1) AFB_1_-lys adduct formation likely occurs exclusively in the liver, as it is the site of both HSA synthesis and the metabolic activation of AFB_1_ by cytochrome P450 enzymes to its reactive carcinogenic metabolite (for which the AFB_1_-lys adduct is a surrogate biomarker) [4]; (2) HSA is a major regulator of oncotic pressure in circulation [25]. 

First, it has been proposed that since hepatic injury or disease can broadly impair hepatic protein synthesis and metabolism, including the metabolism of AFB_1_, HSA concentrations can be used as a surrogate biomarker of HSA synthesis and, ultimately, hepatic function. It is true that HSA synthesis and HSA levels are reduced in severe hepatic or perihepatic disease, such as cirrhosis and gallbladder cancer [18,19,20,26,27]. Additionally, measurements of hepatic metabolic capacity correlate with circulating HSA concentration in persons with chronic liver disease [28,29]. However, while circulating HSA and hepatic function (assessed by antipyrine clearance or similar approaches) are weakly correlated in cases of severe liver disease, HSA is not a sensitive biomarker of changes in hepatic function in these individuals [30,31]. Thus, it is not surprising that HSA is not predictive of hepatic metabolic capacity in persons without disease [28]. In an average adult, a substantial and clinically relevant decrease in HSA concentration (e.g., 20%, from 42 mg/mL to 34 mg/mL; an excursion outside of the reference range) does not necessarily have any association with hepatic metabolic capacity. As a result, HSA normalization would not be effective in accounting for between-person variation in hepatic function.

Plasma volume can be affected by hydration status (through ingestion, excretion, or perspiration), disease (e.g., sepsis), and certain physiological states (e.g., pregnancy). A second rationale for HSA normalization suggests that alterations in plasma volume could increase or decrease the volumetric concentration of circulating AFB_1_-lys adducts, despite a constant level of AFB_1_ exposure and a constant rate of AFB_1_-lys adduct formation. It has been proposed that HSA concentration is similarly altered by changes in plasma volume and thus can be used to adjust for between-person variation in plasma osmolarity, comparable to how creatinine is used to normalize concentrations of urinary biomarkers [32]. However, this hypothesis assumes that HSA concentrations change with plasma osmolarity as a simple function of volumetric dilution or concentration, in parallel with other circulating compounds. In reality, HSA accounts for roughly 80% of oncotic pressure [30], which directly regulates its synthesis [25]. Thus, a decrease in plasma volume will result in a compensatory increase in HSA synthesis. Given this dynamic system (and the movement of HSA between vascular and interstitial spaces [30]), it is inaccurate to use circulating HSA concentration as an independent biomarker of the relative dilution or concentration of plasma. As an example, consider an HSA measurement in an individual that is roughly 20% higher than the population mean (e.g., 50 mg/mL). Interpreted strictly as a volumetric concentrating effect (and ignoring the possibility of inaccurate quantitation by HSA assay), this would require a decrease of roughly 0.6 L plasma volume in the average adult [33,34], enough to induce hypovolemic shock. Such HSA values are not uncommon in clinical and epidemiological data. 

Finally, certain exogenous factors (e.g., nutritional status, infection, hepatic injury or disease) can decrease circulating HSA concentrations. It has been proposed that decreased hepatic HSA production could reduce the availability of HSA as a substrate for the formation of AFB_1_-lys adducts. Assuming this hypothesis is correct, given that circulating HSA concentration is generally reflective of hepatic HSA synthesis [30], the serum/plasma HSA concentration could be used to adjust for between-person variation in hepatic HSA synthesis and downstream AFB_1_-lys adduct formation. However, circulating HSA is not a reliable indicator of nutritional status, except in severe cases of protein malnutrition, such as kwashiorkor [30,35,36]. In addition, decreases in HSA during acute infection is not due to decreased synthesis, but predominantly due to increased vascular permeability and a loss of circulating HSA to the interstitial compartment [30]. 

Moreover, perhaps the strongest case against this rationale for HSA normalization can be derived from the inference that, given essentially any level of aflatoxin exposure, HSA is not a limiting reagent for AFB_1_-lys adduct formation. While HSA synthesis is indeed reduced due to liver disease or injury, even in a setting of extremely high aflatoxin exposure (e.g., 1 μg/day), HSA is present in the liver at a molar ratio of roughly 100,000:1 relative to AFB_1_ (see Appendix A). This calculation is based on the known masses of AFB_1_ and HSA, coupled with experimental data regarding the proportion of ingested AFB_1_ that reaches the liver (~40% [37,38]) and the average rate of hepatic HSA synthesis (~150 mg/kg/day [30]). Thus, HSA is present in in the liver in such stoichiometric excess that, even given a dramatic reduction in synthesis rate due to hepatic disease, HSA would still not be a limiting reagent for AFB_1_-lys adduct formation. 

Thus, HSA normalization: (1) does not or cannot address random technical variance present in AFB_1_-lys measurements, (2) may add more technical variance than the existing between-person biological variance that it is meant to adjust for, and (3) cannot or is not needed to control for between-person biological variance in hepatic metabolism, plasma osmolarity, or HSA synthesis. Furthermore, while AFB_1-_lys internal dose values can be translated to estimates of dietary intake—as we have done in a recent study of mother-child dyads in Bangladesh and Malawi [24]—HSA normalization adds complexity and error to this work due to quantitative biases of various HSA assays used across disparate studies. Coupled with the added time, financial costs, and sample consumption required to perform HSA measurements alongside each AFB_1_-lys determination, there is no compelling basis for the continued use of HSA normalization in AFB_1_-lys adduct dosimetry when using the current state-of-the-art LCMS AFB_1_-lys assay. 

## 4. Conclusions

Overall, the effect of HSA “normalization” in HSA-corrected AFB_1_-lys adduct values appears to be the result of arithmetic transformation and the addition of technical error, rather than a reduction in biological or technical variance. In future work, this conclusion could be further tested using subject-level data on hepatocellular carcinoma incidence and AFB_1_-lys adduct levels, assessing whether raw or HSA-normalized AFB_1_-lys values better recapitulate the well-established causal relationship between this cancer and AFB_1_ exposure, or by comparing measured AFB_1_ intake with raw vs. normalized AFB_1_-lys values. However, given our current results and combined with the added cost, analysis time, sample consumption, and heterogeneity between HSA assays, it seems prudent to abandon the practice of normalizing HSA adducts to the total circulating HSA concentration when measuring any HSA adducts—not only AFB_1_-lys adducts—by isotope-dilution mass spectrometry in serum or plasma. As a result, we have initiated this shift within our own laboratory, recently showing that elevated raw AFB_1_-lys concentrations were significantly associated with the cervical detection of oncogenic strains of HPV in Kenyan women [39] and, separately, revealing seasonal, demographic, and behavioral predictors of aflatoxin exposure in 828 mother-child dyads from Bangladesh and Malawi [24]. Eschewing unnecessary and resource-consuming practices such as the HSA normalization of AFB_1_-lys adducts will allow for more efficient use of precious samples and research funds, while producing superior data.

## 5. Materials and Methods

All HSA and AFB_1_-lys adduct data used for this secondary analysis have been previously peer-reviewed.

### 5.1. Guatemala Study

This study was conducted between May and October of 2016 and was approved by the institutional review boards at the Johns Hopkins Bloomberg School of Public Health and the Institute of Nutrition of Central America and Panama (INCAP). Study details have been published previously [8,40]. All participants provided informed consent. Overall, 461 individuals were recruited from five departments in Guatemala (Quiché, Sololá, Suchitepéquez, Escuintla, and Guatemala). Serum was obtained from 444 individuals, of which 443 had suitable volume for AFB_1_-lys adduct determination by LCMS [5], HSA by ELISA, and HBV and HCV infection status by automated immunoassay, as previously described [8]. Participants were considered for inclusion in the study if they had an HSA measurement, detectable AFB_1_-lys (≥0.01 pg/μL), valid determination of HCV and HBV infection status, and self-reported weekly ethanol consumption. A total of 400 persons without any missing data were available for the present analysis. Individuals were excluded from further analysis on the basis of excessive alcohol use (≥98 g ethanol/7 drinks per week for women or ≥196 g ethanol/14 drinks per week for men; *n* = 11), positive HBV or HCV status (*n* = 54), excessive alcohol use and positive HBV or HCV status (*n* = 4), or missing HBV or HCV status (*n* = 4). The final analytical sample was *n* = 327.

### 5.2. Qidong Community Longitudinal Study

This study was approved by the institutional review boards at the Johns Hopkins Bloomberg School of Public Health and the Qidong Liver Cancer Institute (QDLCI). All study participants provided informed consent. Samples for this study were collected as part of screening protocols for liver cancer early detection and chemoprevention studies in Qidong, People’s Republic of China, dating to the 1980s. From our biorepository, we randomly selected age-matched subsamples of 50 males and 50 females, residing in two neighboring townships (Hezuo and Daxin, ~25 km apart), obtained from screening protocols conducted during the years of 1995, 1999, 2003, 2009, and 2012. Each subsample consisted of a unique set of community residents; subject-level repeated sampling across survey years was not available. Serum samples from the participants were analyzed for AFB_1_-lys by LCMS [5] and HSA by bromocresol purple dye-binding assay (BCP); these results have been published previously [14]. Participants were considered for inclusion if they had both a valid HSA measurement and detectable AFB_1_-lys (≥0.01 pg/μL). Due to the very low rate of detectable AFB_1_-lys in samples from 2012 (7%), subjects from the 2012 sub-sample were excluded from further analysis in the present study. A total of 306 persons from four community screenings (1995, 1999, 2003, and 2009) had AFB_1_-lys and HSA data available for analysis (1995, *n* = 97; 1999, *n* = 92; 2003, *n* = 87; 2009, *n* = 30).

### 5.3. Gallbladder Cancer Case-Control Studies

We have previously reported positive associations between aflatoxin exposure and gallbladder cancer (GBC) in two case-control studies, conducted in the country of Chile [11] and in Shanghai, China [12]. Each study was approved by the institutional review boards at the National Cancer Institute and the respective local study coordinating sites, the Catholic Pontifical University of Santiago, and the Shanghai Cancer Institute, respectively. Details of each study have been published previously [11,12]. In Chile, persons with GBC (with or without the concurrent detection of gallstones) were recruited from hospitals in Santiago, Concepción, and Temuco. Controls consisted of persons with gallstones or community members and were matched to GBC cases by age and sex. Controls were additionally matched to GBC cases by location of enrollment, while persons with gallstones were matched to GBC cases diagnosed at the same referring hospital. Hereafter, “controls” will be used to refer to either persons with gallstones or community participants. For the study conducted in China, persons with GBC (with or without the concurrent detection of gallstones) were referred for enrollment from the Shanghai Cancer Institute and 42 collaborating Shanghai hospitals from June 1997 through May 2001. Persons undergoing medical treatment or cholecystectomy for gallstones at the same hospital as an enrolled GBC case were matched by sex and age (within five years) and referred for study enrollment as controls. In both the Chilean and the Chinese studies, serum samples were analyzed for AFB_1_-lys by LCMS [5] and HSA by bromocresol purple dye-binding assay (BCP); these results have been published previously [11,12]. Participants were considered for inclusion if they had both a valid HSA measurement and detectable AFB_1_-lys (≥0.01 pg/μL); in prior publications [11,12], only participants with normalized AFB_1_-lys levels ≥0.05 pg/mg HSA were included for analysis. Data were pooled across both study sites, resulting in a final analytical sample size of *n* = 130 persons with GBC (Chile, *n* = 23; China, *n* = 107) and *n* = 85 controls (Chile, *n* = 8 persons with gallstones and *n* = 10 community controls; China, *n* = 67 persons with gallstones). The combined data set was analyzed as one study, referred to hereafter as the “GBC case-control” study.

### 5.4. Data Analysis

All analyses were conducted using SAS 9.4 (SAS Institute, Cary, NC). “Raw” AFB_1_-lys adduct refers to serum AFB_1_-lys—albumin adduct concentration—in the units of pg AFB_1_-lys adduct/mL serum. “HSA-normalized” or “normalized” AFB_1_-lys adduct levels refers to the raw AFB_1_-lys adduct level divided by the total HSA concentration (in mg/mL), resulting in the final units of pg AFB_1_-lys adduct/mg HSA. Within the GBC case-control study, the distributions of HSA, raw AFB_1_-lys adduct levels, and HSA-normalized AFB_1_-lys concentrations were compared in cases and controls using the non-parametric Wilcoxon signed-rank test using the PROC NPAR1WAY procedure. 

Linear regression models were used to assess the relative contributions of HSA, raw AFB_1_-lys levels, and various demographic parameters to the total observed variance in AFB_1_-lys internal dose in the Guatemalan dataset. Multiple models were constructed, with either raw AFB_1_-lys or HSA-normalized AFB_1_-lys as the dependent variable and the independent variables consisting of HSA, raw AFB_1_-lys, and various demographic factors (sex, urban/rural residence, educational attainment, and income) that we have found to be associated with aflatoxin exposure in Guatemala [8]. In all models, slope estimates were used to assess the importance of each parameter in the model, and the adjusted R^2^ was used to assess the proportion of explained variation (vs. coefficient of determination) of the AFB_1-_lys internal dose.

A Monte Carlo simulation approach [41] was used to construct simulated data sets of HSA and raw AFB_1_-lys adduct concentrations, modeled on the empirical data from each study. Specifically, within each study, the empirically observed mean and standard deviation values for HSA concentration were used to construct 20 normally or lognormally distributed simulated samples of 1000 randomly generated observations each, using the RDRAND function in SAS and a seed value of 0 [42]; these 20 independent samples were then combined into a single dataset of 20,000 simulated observations. A 20,000-observation simulated dataset was similarly constructed for the raw AFB_1_-lys adduct concentrations using the empirically observed raw AFB_1_-lys adduct mean and standard deviation. Next, the randomly generated HSA and AFB_1_-lys values in each dataset were sequentially paired, without replacement, to create a third dataset of simulated study participants, and the HSA-normalized AFB_1_-lys adduct values were calculated for each simulated participant. Within each study (Guatemala, China, GBC case-control), these simulated datasets were constructed using distribution parameters drawn from the entire sample, independent of the year of sample collection (China), disease status (GBC case-control), or study site (GBC case-control). Thus, for each of the three studies, we created a unique 20,000-observation simulated dataset with distribution parameters essentially identical to the empirical data, but where intra-person relationships between raw and HSA-normalized AFB_1_-lys adduct levels were the result of arithmetic transformation and random variation rather than biology. 

Linear regression was used to assess the relationship between raw AFB_1_-lys adduct levels and HSA-normalized adduct levels. Residuals were obtained from linear regression models in which the raw AFB_1_-lys adduct level was the lone independent variable and the HSA-normalized AFB_1_-lys concentration was the dependent variable to be predicted. Thus, the regression residuals constitute the differences between the empirically observed HSA-normalized AFB_1_-lys adduct level (pg/mg HSA) at a given concentration of the raw AFB_1_-lys adduct (pg/mL serum or plasma) vs. the predicted HSA-normalized AFB_1_-lys adduct level at the same concentration of the raw AFB_1_-lys adduct. In other words, the residuals were visualized as the ordinate (*y*-axis) deviation of an individual observation from the best-fit linear regression line describing the relationship between raw and HSA-normalized adduct levels. To compare residuals across five orders of magnitude of raw AFB_1_-lys adduct levels, Studentized regression residuals were standardized as Z-scores ($z=\left[\;x-\bar{x}\;\right]\;/\;s$). Simulated data were analyzed in the same fashion as empirical observations and distributions of empirical and simulated residuals were compared. 

## Figures and Tables

**Table 1:** Multiple linear regression of AFB_1_-lys adduct concentrations in the Guatemalan study. ^1^.

	^1^ Normalized (pg/mg HSA)						^1^ Raw (pg/mL Serum)		
	Model 1			Model 2			Model 1		
Parameter	Estimate (SE)	*p*	R^2^	Estimate (SE)	*p*	R^2^	Estimate (SE)	*p*	R^2^
Intercept	3.086 (0.556)	<0.0001	0.124	−1.595 (0.038)	<0.0001	0.976	3.086 (0.556)	<0.0001	0.083
^2^ Raw AFB_1_-lys	-	-		0.999 (0.009)	<0.0001		-	-	
^2^ HSA	−0.951 (0.336)	0.005		-	-		0.049 (0.336)	0.883	
^3^ Male	−0.140 (0.057)	0.016		−0.003 (0.010)	0.761		−0.140 (0.057)	0.016	
^4^ Urban	−0.194 (0.063)	0.002		−0.051 (0.010)	<0.0001		−0.194 (0.063)	0.002	
^5^ Education	−0.004 (0.008)	0.668		−0.001 (0.001)	0.602		−0.004 (0.008)	0.668	
^6^ Income	−0.097 (0.033)	0.003		0.002 (0.006)	0.674		−0.097 (0.033)	0.003	

^1^*n* = 325, two participants had missing data for income. ^2^ AFB1-lys and HSA concentrations were log_10_-transformed before analysis. ^3^ vs. female. ^4^ vs. rural. ^5^ per year of education. ^6^ per income quintile.

## Data Availability

SAS code and raw data will be made available upon request.

## Supplementary Materials

The following supporting information can be downloaded at: https://www.mdpi.com/article/10.3390/toxins14030162/s1, Figure S1: HSA, raw AFB_1_-lys, and normalized AFB_1_-lys levels in each gallbladder cancer (GBC) case-control study.

## Appendix A

Ratio of hepatic HSA/AFB_1_ in 70 kg adult, assuming:

- 1 μg daily AFB_1_ dose, or 3.20 nmol/day (AFB_1_ mol. weight, 312.3 g/mol);
- ~40% of ingested AFB_1_ reaches the liver via portal circulation [37,38];
- Hepatic HSA synthesis rate of 150 mg/kg/day [31], or 2.26 μmol/kg/day (HSA mol. weight, 66,472 g/mol)

$$\frac{\left(2.26{\;\mu \mathrm{mol}\;\mathrm{HSA}*\mathrm{kg}}^{-1}{*\;\mathrm{day}}^{-1}\right)}{\left(0.00320{\;\mu \mathrm{mol}\;\mathrm{AFB}}_{1}*0.40*70{\;\mathrm{kg}}^{-1}{*\mathrm{day}}^{-1}\right)}=\frac{123,594\;\mathrm{mol}\;\mathrm{HSA}}{1{\;\mathrm{mol}\;\mathrm{AFB}}_{1}}$$
