# Supplementary material for: Assessing the Validity of Normalizing Aflatoxin B1-Lysine Albumin Adduct Biomarker Measurements to Total Serum Albumin Concentration across Multiple Human Population Studies
Source: Toxins (Basel). 2022 Feb 23;14(3):162. doi: 10.3390/toxins14030162 (PMC8954427; doi:10.3390/toxins14030162)
Supplement: Supplementary file 1 [file toxins-14-00162-s001.zip › toxins-1577522-supplementary.pdf]

toxins

MDPI

Article

Assessing the Validity of Normalizing Aflatoxin B1-Lysine Albumin Adduct Biomarker Measurements to Total Serum Albumin Concentration across Multiple Human Population Studies

Joshua W. Smith, Derek K. Ng, Christian S. Alvarez, Patricia A. Egner, Sean M. Burke, Jian-Guo Chen, Thomas W. Kensler, Jill Koshiol, Alvaro Rivera-Andrade, María F. Kroker-Lobos, Manuel Ramírez-Zea, Katherine A. McGlynn and John D. Groopman

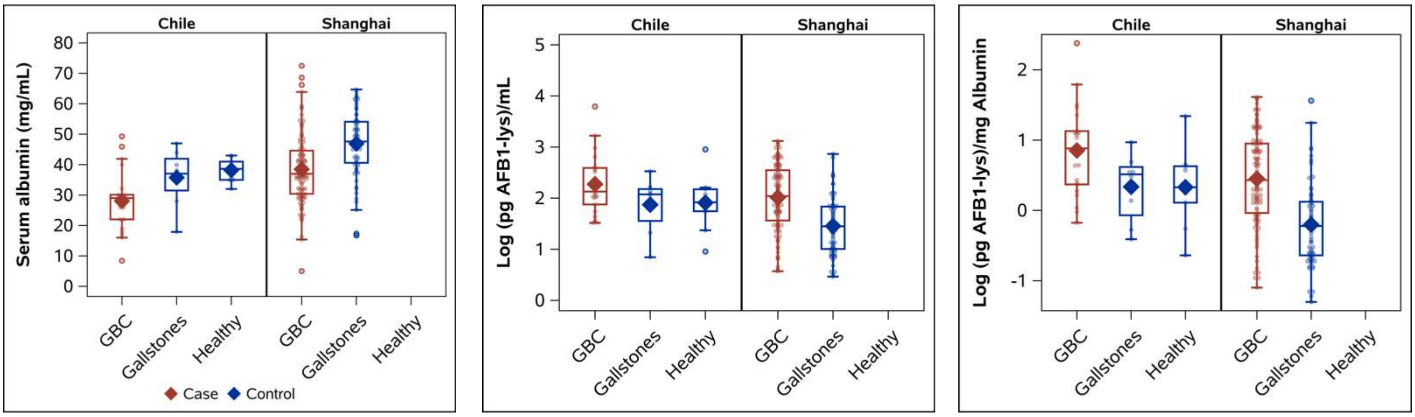

Figure S1. HSA, raw AFB1-lys, and normalized AFB1-lys levels in each gallbladder cancer (GBC) case-control study.
